# Morpho-phylogenic characterization of *Neonectria candida* as a causal agent of a postharvest rot of pome fruit in the U.S. Pacific Northwest

**DOI:** 10.3389/fpls.2025.1661560

**Published:** 2025-09-11

**Authors:** Joseph K. Mellow, Arild R. Arifin, Achour Amiri

**Affiliations:** Department of Plant Pathology, Washington State University, Tree Fruit Research and Extension Center, Wenatchee, WA, United States

**Keywords:** *Neonectria* spp., cylindrocarpon, pome fruit, virulence, fungal pathogen, postharvest, multilocus sequence analysis

## Abstract

Neonectria fruit rot (NFR) is primarily attributed to *Neonectria ditissima*, the causal agent of European canker, in many apple-growing regions globally. Between 2017 and 2019, NFR-like symptoms were observed in several surveyed apple and pear packinghouses in Washington State and Oregon. In this study, 52 *Neonectria* isolates were characterized using a multilocus sequence analysis (MLSA), pathogenicity assays, and morphological traits across various growth media and photoperiods. MLSA analysis of four DNA regions, i.e., *β-TUB, TEF1*, LSU, and ITS rDNA, identified the pathogen as *Neonectria candida* (syn. *Neonectria ramulariae*, anamorph *Cylindrocarpon obtusiusculum*). All the 52 *N. candida* isolates formed a distinct clade from *N. ditissima* and other *Neonectria* spp. with a Bayesian interference posterior probability of 0.98. Fruit pathogenicity assays showed that *N. candida* isolates caused light brown lesions on ‘Fuji’ apples and ‘Green d’Anjou’ pears both at room temperature (22°C) and cold storage (0.5 and 1.5°C), with NFR incidences ranging from 6 to 100% after 15 days to four months. *N. candida* isolates grew and sporulated profusely under a multitude of nutrient and photoperiod conditions *in vitro*. This study is a foundational step towards species identification and understanding the biology and epidemiology of NFR to support the development of effective management approaches.

## 1 Introduction

Over 100 fungal species have been reported to infect apples and pears at different phenological stages before harvest and during storage (Sutton et al., 2014). In the U.S. Pacific Northwest (PNW) and other pome fruit growing regions, the most economically import fungal pathogens include *Penicillium* spp. (Sanderson and Spotts, 1995), *Botrytis* spp. (Amiri and Ali, 2016), *Neofabraea* spp. (Henriquez et al., 2007), *Mucor* spp. (Bertrand and Saulie-Carter, 1980), *Alternaria* spp. (Filajdić and Sutton, 1991), *Cladosporium* spp. (Virginia et al., 2021)*, Phacidiopycnis* spp. (Xiao and Boal, 2004), *Sphaeropsis* spp. (Kim and Xiao, 2008), *Lambertella corni-marris* (Wiseman et al., 2015), *Colletotrichum* spp. (Khodadadi et al., 2020), and *Neonectria* spp. (Holthusen and Weber, 2021). Beyond direct losses resulting from decay, some of these pathogens can taint processed pome fruit products with mycotoxins (Brian et al., 1956; Zhong et al., 2018) or trigger quarantine restrictions that block access to export markets (Hansen, 2014).

Among the above-mentioned pathogens, *Neonectria* spp. have a wide host range and can infect more than 60 tree and shrub species across the Myrtaceae, Pinaceae, Proteaceae, and Rosaceae families, producing a wide range of symptoms (Chaverri et al., 2011; Hirooka et al., 2013). In the Rosaceae, *Neonectria* and its anamorph *Cylindrocarpon* spp. cause cankers and dieback on trunks and young shoots, weakening them and reducing yields if unmanaged (Ghasemkhani et al., 2016). *Neonectria* belongs to the Nectriaceae, characterized by brightly colored, uniloculate perithecia (Tulasne and Tulasne, 1865). Species limits are complicated, prompting multilocus phylogenetics. Chaverri et al. (2011) analyzed 66 isolates with six loci (LSU, ITS*, RPB1, RPB2, β-tubulin, TEF1, ACT*), stabilizing *Cylindrocarpon* taxonomy and placing *N. ditissima, N. candida*, and *N. fuckeliana* in *Neonectria sensu stricto.* Among these species, *N. ditissima, N. punicea, and N. candida* have been identified as causal agents of Neonectria fruit rot (NFR) in pome fruits grown in England (Xu and Robinson, 2010), Netherlands (Wenneker et al., 2016), and China (Wu et al., 2022). NFR symptoms are seldom visible in the orchard, even in humid areas, and are virtually undetected in the arid central-Washington climate. Instead, circular, slightly sunken, light- to dark-brown lesions with firm to soft texture usually appear after several months of cold storage (Edwards, 2006; Xu and Robinson, 2010). Yellow- to white-pustulate sporulation follows as the rot advances (Munson, 1939). Early NFR caused by *N. ditissima* closely mimics bull’s-eye rot caused by *Neofabraea* spp., leading to frequent misdiagnosis (Xu and Robinson, 2010) and potential fruit rejection during transit or retail.

Recent reports have expanded the NFR complex, i.e., *N. punicea* on ‘Red Delicious’ and ‘Fuji’ apples in China (Wu et al., 2022) and *N. candida* (syn. *N. ramulariae*, anamorph *Cylindrocarpon obtusiusculum*) on ‘Conference’ pears in the Netherlands (Wenneker et al., 2016). In accordance with the “one fungus, one name” (Hawksworth, 2011), the name *Neonectria candida* is used instead of its synonyms or anamorphic names. Infections of the NFR originate through natural openings (stem-end, calyx-end, lenticels) or harvest wounds. Besides infecting pears, *N. punicea* and *N. candida* were reported to cause seed rot, stem dieback, or canker diseases on a variety of forest trees (Hirooka et al., 2012, 2013; Jankowiak et al., 2016; Karadžić et al., 2020). While the ability of *N. ditissima*, *N. punicea*, and *N. candida* to infect fruit is documented elsewhere, their capacity to produce cankers and fruit rot in PNW orchards, and their local distribution and epidemiology, remain largely unexplored. In large surveys conducted between 2016 and 2019 in several apple and pear packinghouses in Washington State and Oregon, a postharvest decay resembling Neonectria-like disease, was observed in fruit originating from several orchards (Amiri and Ali, 2016; Mellow and Amiri, 2023).

We conducted this study to address three primary objectives: i) identify the species causing Neonectria fruit rot (NFR) in pome fruit from the PNW through multi-locus phylogenetic analysis; ii) characterize the morphology of the isolates *in vitro* under varying media and photoperiod conditions; and iii) evaluate the pathogenicity and virulence of these species on detached apple and pear fruit.

## 2 Materials and methods

### 2.1 Collection, culturing, and characterization of *Neonectria* isolates

**Figure 1:**
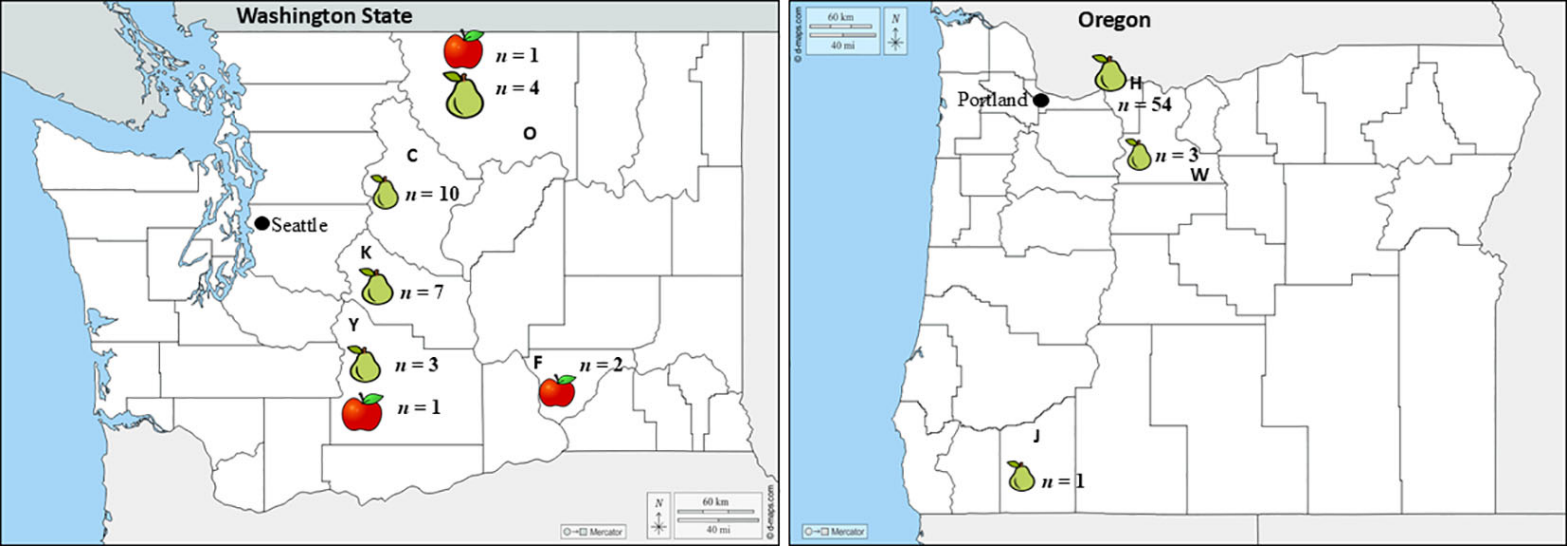
Geographic origin (state, county) of the isolates collected by host (apple or pear) in Washington and Oregon. Bold letters C, F, K, O, and Y represent Chelan, Franklin, Grant, Kittitas, Okanogan and Yakima counties in Washington, respectively, whereas H, J, and W indicates the Hood River and Jackson, Wasco counties in Oregon, respectively. The *n* next to each host in each county indicates the number of isolates collected.

Decayed apples and pears were collected on the packing line after six to eight months of storage under standard commercial conditions between 2017 and 2019 from multiples apple and pear packinghouses in Washington State (WA) and Oregon (OR), United States (**Figure 1**). Fruits were transported in clean clamshells to the laboratory and decay types were identified based on key morphological characteristics (Amiri et al., 2017; Ali et al., 2018). Pure fungal cultures were made from each fruit on General Isolation (GI) medium consisting of half-strength potato dextrose agar (PDA) (Hardy Diagnostics, Santa Maria, CA), 7.5 g of bacto agar (Difco Laboratories, Sparks, MD), in 1L of distilled water supplemented with 200 mg/L streptomycin sulfate (Amresco, Solon, OH) and 100 mg/L ampicillin (MP Biomedicals, Solon, OH) added post-autoclave. The decayed fruits were cut open using an ethanol-sterilized knife, and a small piece of flesh at the margin between decayed and healthy fruit tissue was cut and transferred to GI plates (Amiri et al., 2017). Cultures were incubated at 22 °C for 10 days, and fungal isolates were characterized based on known colony morphology, and by spore shape and conidiophore structure according to Dugan (2006). Pure cultures were then preserved as mycelial plugs in 20% glycerol and stored at -80 °C in the Pathology Lab Washington State University (WSU) - Tree Fruit Research and Extension Center (TFREC), Wenatchee, WA. Conidial and conidiophore morphology was assessed microscopically for representative isolates. After 28 days on PDA, each culture was washed with 10 mL sterile water, scraped, and the resulting suspension filtered through cheesecloth; spore concentration was then determined with a hemacytometer on a Zeiss Axioskop microscope (Zeiss, Oberkochen, Germany). A 50 µl droplet of each suspension was placed on a PDA-coated slide, incubated at 23°C under a 12 hr photoperiod for three days (Hua’an et al., 1991), and examined with Olympus BX53 compound (Olympus, Central Valley, PA) and Zeiss Stemi 508 dissecting microscopes, with images captured on an AmScope camera (AmScope, Los Angeles, CA).

### 2.2 Molecular characterization of *Neonectria* isolates

The Neonectria-like isolates (**Table 1**) were revived from -80°C storage on PDA and incubated for 14 days at 22°C. Peripheral mycelial plugs were transferred to 50 mL of sterile potato dextrose broth in flasks and shaken at 160 rpm for 5 days at 22°C. The resulting mycelia were harvested on 8 µm Whatman filter paper with a Büchner funnel, transferred to 1.5 mL tubes, and stored at -20°C. For DNA extraction, ~100 mg of thawed mycelium was combined with 200 mg of glass-silica beads in a 1.5 mL tube and homogenized in a FastPrep-24™ grinder (MP Biomedicals, Irvine, CA). Genomic DNA was then isolated using the alkaline-lysis method of Raeder and Broda (1985), resuspended in 100 µL sterile distilled water, quantified with a NanoDrop 2000 (Thermo Fisher Scientific, Waltham, MA), and stored at -20°C.

**Table 1:** Details about the isolates, species identifications and accession numbers used for the phylogenetic analysis.

Isolate	Year	State	Host	Cultivar	Species identification based on				GenBank Accession number			
					*β-TUB^b^*	*TEF1*	*LSU*	*ITS rDNA*	*β-TUB*	*TEF1*	*LSU*	*ITS rDNA*
17-1152	2017	WA	Pear	Green d'Anjou	*N. candida*	*N. candida*	*N. candida*	*N. candida*	PP024667	PP175934	PP101133	OR882918
17-1160	2017	WA	Pear	Green d'Anjou	*N. candida*	*N. punicea*	*N. candida*	*N. candida*	PP084323	PP216927	PP101169	OR882919
17-1161	2017	WA	Pear	Green d'Anjou	*N. candida*	*N. candida*	*N. candida*	*N. candida*	PP084319	PP216928	PP101175	OR882920
17-1162	2017	WA	Pear	Green d'Anjou	*N. candida*	*N. candida*	*N. candida*	*N. candida*	PP034667	PP216929	PP101182	OR882921
17-1165	2017	WA	Pear	Green d'Anjou	*N. candida*	*N. candida*	*N. candida*	*N. candida*	PP077006	PP175938	PP101157	OR882922
17-1168	2017	WA	Pear	Green d'Anjou	*N. candida*	*N. candida*	*N. candida*	*N. candida*	PP034668	PP236396	PP101174	OR882923
17-1176	2017	OR	Pear	Green d'Anjou	*N. candida*	*N. candida*	*N. candida*	*N. candida*	PP034656	PP236397	PP101135	OR882924
**17-1177** ^a^	**2017**	**OR**	**Pear**	**Green d'Anjou**	*N. candida*	*N. punicea*	*N. candida*	*N. candida*	PP215349	PP230547	PP101166	OR882925
17-1183	2017	WA	Pear	Bosc	*N. candida*	*N. punicea*	*N. candida*	*N. candida*	PP097435	PP230553	PP101147	OR882926
17-1194	2017	WA	Pear	Bosc	*N. candida*	*N. punicea*	*N. candida*	*N. candida*	PP034669	PP236395	PP101165	OR882927
17-669B	2017	OR	Pear	Bosc	*N. candida*	*N. candida*	*N. candida*	*N. candida*	PP034657	PP236401	PP101142	OR882928
**17-691**	**2017**	**WA**	**Apple**	**Gala**	*N. candida*	*N. candida*	*N. candida*	*N. candida*	PP034655	PP236387	PP101141	OR882929
**17-703**	**2017**	**WA**	**Apple**	**Gala**	*N. candida*	*N. candida*	*N. candida*	*N. candida*	PP034662	PP175935	PP101173	OR882930
17-705A	2017	WA	Apple	Gala	*N. candida*	*N. candida*	*N. candida*	*N. candida*	PP215347	PP236394	PP101137	OR882931
**17-764**	**2017**	**OR**	**Pear**	**Green d'Anjou**	*N. candida*	*N. candida*	*N. candida*	*N. candida*	PP034665	PP216926	PP101145	OR882932
17-782A	2017	OR	Pear	Red d'Anjou	*N. candida*	*N. punicea*	*N. candida*	*N. candida*	PP130546	PP236392	PP101172	OR882933
17-782B	2017	OR	Pear	Red d'Anjou	*N. candida*	*N. punicea*	*N. candida*	*N. candida*	PP097436	PP230551	PP101180	OR882934
17-795	2017	OR	Pear	Red d'Anjou	*N. candida*	*N. punicea*	*N. candida*	*N. candida*	PP215348	PP230543	PP101140	OR882935
17-796	2017	OR	Pear	Red d'Anjou	*N. candida*	*N. punicea*	*N. candida*	*N. candida*	PP112386	PP230541	PP101177	OR882936
17-799	2017	OR	Pear	Green d'Anjou	*N. candida*	*Nectria balansae*	*N. candida*	*N. candida*	PP112382	PP230546	PP101132	OR882937
17-805	2017	OR	Pear	Bosc	*N. candida*	*Nectria balansae*	*Nectria balansae*	*-*	PP215350	PP230552	PP101152	-
17-810	2017	WA	Pear	Green d'Anjou	*N. candida*	*N. candida*	*N. candida*	*N. candida*	PP034658	PP216922	PP101179	OR882938
17-863	2017	WA	Pear	Green d'Anjou	*N. candida*	*N. candida*	*N. candida*	*N. candida*	PP077007	PP216915	PP101146	OR882939
17-911	2017	WA	Pear	Green d'Anjou	*N. candida*	*N. candida*	*N. candida*	*N. candida*	PP084321	PP175937	PP101171	OR882940
17-912	2017	WA	Pear	Green d'Anjou	*N. candida*	*N. candida*	*N. candida*	*N. candida*	PP034663	PP236388	PP101158	OR882941
17-949	2017	WA	Pear	Green d'Anjou	*N. candida*	*N. candida*	*N. candida*	*N. candida*	PP215346	PP216924	PP101159	OR882942
17-960	2017	WA	Pear	Green d'Anjou	*N. candida*	*N. candida*	*N. candida*	*N. candida*	PP077004	PP216923	PP101143	OR882943
17-968	2017	WA	Pear	Green d'Anjou	*N. candida*	*N. punicea*	*N. candida*	*N. candida*	PP097437	PP230544	PP101185	OR882944
18-626	2018	OR	Pear	Red d'Anjou	*N. candida*	*N. candida*	*N. candida*	*N. candida*	-	PP236399	PP101183	OR882945
18-627	2018	OR	Pear	Red d'Anjou	*N. candida*	*N. punicea*	*N. candida*	*N. candida*	PP034671	PP230548	PP101136	OR882946
18-628	2018	OR	Pear	Red d'Anjou	*N. candida*	*N. candida*	*N. candida*	*N. candida*	PP034661	PP216918	PP101170	OR882947
18-629	2018	OR	Pear	Red d'Anjou	*N. candida*	*N. candida*	*N. candida*	*N. candida*	PP130544	PP216919	PP101184	OR882948
18-630	2018	OR	Pear	Red d'Anjou	*N. candida*	*N. candida*	*N. candida*	*N. candida*	PP084324	PP216930	PP101155	OR882949
18-631	2018	OR	Pear	Red d'Anjou	*N. candida*	*N. candida*	*N. candida*	*N. candida*	PP130543	PP236400	PP101163	OR882950
18-635	2018	OR	Pear	Green d'Anjou	*N. candida*	*N. punicea*	*N. candida*	*N. candida*	PP112385	PP216925	PP101134	OR882951
18-636	2018	OR	Pear	Green d'Anjou	*N. candida*	*N. punicea*	*N. candida*	*N. candida*	PP097439	PP230545	PP101160	OR882952
18-645	2018	OR	Pear	Green d'Anjou	*-*	*N. punicea*	*N. candida*	*N. candida*	-	PP230550	PP101154	OR882953
18-661	2018	OR	Pear	Bosc	*N. candida*	*N. candida*	*N. candida*	*N. candida*	PP034664	PP216920	PP101181	OR882954
18-664	2018	OR	Pear	Bosc	*N. candida*	*N. punicea*	*N. candida*	*N. candida*	PP112387	PP230549	PP101144	OR882955
18-672	2018	OR	Pear	Red d'Anjou	*N. candida*	*N. candida*	*N. candida*	*N. candida*	PP034670	PP236393	PP101178	OR882956
**18-673**	**2018**	**OR**	**Pear**	**Red d'Anjou**	*N. candida*	*N. candida*	*N. candida*	*N. candida*	PP084320	PP216917	PP101167	OR882957
18-674	2018	OR	Pear	Green d'Anjou	*N. candida*	*N. punicea*	*N. candida*	*N. candida*	PP112383	PP230542	PP101148	OR882958
18-678	2018	OR	Pear	Green d'Anjou	*N. candida*	*N. punicea*	*N. candida*	*N. candida*	PP097438	PP236391	PP101138	OR882959
18-679	2018	OR	Pear	Green d'Anjou	*N. candida*	*N. candida*	*N. candida*	*N. candida*	PP130542	PP236398	PP101161	OR882960
18-682	2018	OR	Pear	Bosc	*N. candida*	*N. punicea*	*N. candida*	*N. candida*	PP112384	PP236390	PP101164	OR882961
18-684	2018	OR	Pear	Green d'Anjou	*N. candida*	*N. candida*	*N. candida*	*N. candida*	PP034666	PP175936	PP101162	OR882962
**19-004**	**2019**	**WA**	**Apple**	**Koru**	*N. candida*	*N. punicea*	*N. candida*	*N. candida*	-	PP236403	PP101139	OR882963
19-17	2019	WA	Pear	Green d'Anjou	*N. candida*	*N. candida*	*N. candida*	*N. candida*	PP034659	PP216916	PP101176	OR882964
19-18	2019	WA	Pear	Green d'Anjou	*N. candida*	*N. candida*	*N. candida*	*N. candida*	PP077005	PP236402	PP101168	OR882965
19-19	2019	WA	Pear	Green d'Anjou	*N. candida*	*N. candida*	*N. candida*	*N. candida*	PP084322	PP236404	PP101186	OR882966
19-20	2019	WA	Pear	Green d'Anjou	*N. candida*	*N. punicea*	*N. candida*	*N. candida*	PP034660	PP236389	PP101156	OR882967
19-21	2019	WA	Pear	Green d'Anjou	*-*	*N. candida*	*N. candida*	*N. candida*	-	PP216921	PP101153	OR882968

a Bolded isolates are those used for *in vitro* and fruit tests.

b *β-TUB*, *TEF1*, *LSU* and *ITS*, indicate beta tubulin, translation elongation factor-1 alpha, large ribosomal RNA subunit, and ITS rDNA, respectively.

Molecular characterization of isolates was performed using multilocus sequence analysis (MLSA) of four DNA regions known to effectively distinguish Nectriaceae species (Crous et al., 2009; Hirooka et al., 2013; Lombard et al., 2015). The loci included the internal transcribed spacer ribosomal DNA (ITS rDNA [ITS1-5.8S-ITS2] (White et al., 1990), beta-tubulin (*β-TUB*) (Crous et al., 2004; O’Donnell and Cigelnik, 1997), translation elongation factor-1 alpha (*TEF1*) (Carbone and Kohn, 1999; Rehner and Buckley, 2005), and the large ribosomal RNA subunit (LSU) (Vilgalys and Hester, 1990; Crous et al., 2009).

Polymerase chain reaction (PCR) amplifications were performed in 25 µL reactions containing 12.5 µL of EconoTaq PLUS Green 2x Master Mix (Lucigen, Middleton, WI), 1 µL of each 10 μM primer (**Table 2**), 0.2 µL of dimethyl sulfoxide (DMSO), 1.2 µL of bovine serum albumin (BSA), 9.1 µL of nuclease-free water and 1 µL of 20 ng/µL of DNA template. Reactions were conducted using a BIO-RAD T100 thermal cycler (Bio-Rad Laboratories, Hercules, CA) under thermocycling conditions optimized for each primer pair, as previously described (Crous et al., 2004; O’Donnell and Cigelnik, 1997). PCR products were separated by electrophoresis on 1% agarose gels (Green BioResearch LLC, Baton Rouge, LA) in 1× Tris-acetate-EDTA (TAE) buffer, stained with GelRed (Biotium Inc., Fremont, CA), and visualized utilizing a UVP GelDoc-It 130 Imaging System (Analytik Jena U.S., Upland, CA). Successful amplicons were purified using the Wizard SV Gel and PCR Clean-Up System (Promega Corporation, Madison, WI), following the manufacturer’s protocol. Purified products were sequenced bidirectionally at Retrogen, Inc. (San Diego, CA). Raw sequences were trimmed, assembled, and aligned using Geneious Prime v2021.0.3 (Kearse et al., 2012). Final sequences were submitted to the National Center for Biotechnology Information (NCBI) GenBank, and corresponding accession numbers are listed in **Table 1**.

**Table 2:** Primers and their sequences used in this study.

Target region	Primer	DNA Sequence 5’ to 3’	Thermocycler and Primer Reference
Internal transcribed spacer	*ITS5*	GGA AGT AAA AGT CGT AAC AAG G	White et al., 1990
	*ITS4*	TCC TCC GCT TAT TGA TAT GC	White et al., 1990
β-tubulin	*Btub T1F*	AAC ATG CGT GAG ATT GTA AGT	O’Donnell and Cigelnik, 1997
	*Btub CYLTUB1R*	AGT TGT CGG GAC GGA AGA G	Crous et al., 2004
Translation elongation factor	*Tef1-728F*	CAT CGA GAA GTT CGA GAA AGG	Carbone and Kohn, 1999
	*Tef1-1567R*	ACH GTA CCR ATA CCA CCS ATC TT	Hirooka et al., 2013
Large Subunit ribonucleic acid	*LSU-1Fd*	GRA TCA GGT AGG RAT ACC CG	Crous et al., 2009
	*LR5*	ATC CTG AGG GAA ACT TC	Vilgalys and Hester, 1990

### 2.3 Phylogenetic analyses

Single-locus (ITS rDNA, *β-tubulin*, *TEF1-α*, and LSU) and four-locus concatenated phylogenies were inferred for all 52 *Neonectria* isolates. Homologous reference sequences and outgroups (**Supplementary Table S1**) were downloaded from GenBank and aligned using Clustal Omega (Sievers and Higgins, 2014) through Geneious Prime v2021.0.3. Phylogenetic trees were inferred by Maximum-likelihood (ML) using RAxML v8.2.11 (Stamatakis, 2014) with 1,000 rapid bootstrap replicates. In addition, Bayesian inference (BI) trees were generated using MrBayes v3.2.6 (Ronquist and Huelsenbeck, 2012; Ronquist et al., 2012). BI trees were run for 2 million generations and trees were sampled every 1,000 generations using the GTR + G4 model through Geneious Prime v2021.0.3. Convergence was confirmed by an average split-frequency < 0.01 and ESS > 200 (Tracer v1.7). Trees were visualized in FigTree v1.4 (http://tree.bio.ed.ac.uk/software/figtree/). Nodes were considered robust when bootstrap percentages and posterior probabilities were >70% and >0.70, respectively. Topological congruence among the four loci was tested with the partition homogeneity test in PAUP* v4.0a169 (Swofford, 2003) using pairwise homogeneity test (PHT) with heuristic search (100 replicates, *P* < 0.01). To gauge species boundaries, pairwise ITS divergences were calculated in Geneious; isolates differing by < 3% were treated as conspecific (Nilsson et al., 2008; Schoch et al., 2011).

### 2.4 Mycelial growth and sporulation on different media under different photoperiod conditions

From the total of 52 *Neonectria* isolates characterized to the species level (**Table 1**), six representative isolates (each three isolates originated from apple and pear) were selected for detailed physiological assays. Radial growth, sporulation, and micro-/macroconidium production were assessed on PDA, apple-juice agar (AJA; 200 mL organic Fuji juice + 18 g agar + 800 mL H_2_O), V8 agar (V8A), and low-nutrient agar (LNA, Gerlach and Nirenberg, 1982) at 23 °C under three photoperiods: 12 h light/12 h dark, continuous darkness, and continuous light. A 5-mm plug was cut from 14-day PDA cultures of each isolate and placed mycelium-down on each medium (three replicates per isolate × medium × light regime). Colony diameters were measured along two perpendicular axes on days 7, 14, 21, and 28 days and growth rates (mm/day) were calculated from the 14–21-day interval. Plates were photographed (Canon EOS 30D) at each measurement point. After 28 days, conidial suspensions were prepared from the same plates as described above, and micro- and macroconidia were quantified and imaged microscopically following Chaverri et al. (2011). The trial was conducted twice.

### 2.5 Pathogenicity and virulence assays

Pathogenicity and virulence of six *Neonectria* isolates (**Table 1**) were evaluated on ‘Green d’Anjou’ pears and ‘Fuji’ apples harvested at commercial maturity in September and October 2022, respectively, from an experimental orchard in Rock Island, WA. These cultivars were selected because of their importance in commercial orchards in the PNW. Fruits were surface-disinfected in 1% sodium hypochlorite for 2 min, rinsed twice with tap water, and air-dried. Four equatorial wounds (3 mm diameter, 4 mm deep) were made per fruit, and each wound was inoculated with 30 µL of a 1 × 10^5^ conidia/mL suspension of the appropriate isolate. For each isolate, 72 apples and 72 pears were arranged in a randomized complete-block design: two lots of 36 fruit (four replicates of nine). One lot was incubated at 22°C and assessed weekly for 28 days; the second lot was stored at 1.5°C (‘Fuji’) or 0.5°C (‘Green d’Anjou’) and assessed monthly for four months. Disease incidence was recorded as the proportion of wounds exhibiting decay, and disease severity as lesion diameter (average of two perpendicular measurements). To confirm the symptoms were caused by the inoculated pathogen, Koch’s postulates were completed by re-isolation on representative lesions from fruit held 28 days at 22°C or four months at cold temperatures as described above for the initial isolation.

### 2.6 Data analyses

Growth rate and conidial production were analyzed with a three-way ANOVA in which culture medium, light regime, and assessment interval served as fixed factors and isolate identity was treated as a random factor. Normal distribution of the data was assessed using the Shapiro-Wilk test. When the overall F-test was significant (α = 0.05), the means were separated with Student’s *t*-tests. For mycelial growth, *post-hoc* contrasts between every medium × light combination were evaluated. For the pathogenicity assay, lesion diameter (mm) and disease incidence were subjected to separate three-way ANOVAs for each host species, using storage temperature and inspection time as additional factors. Mean separation again employed Student’s *t*-tests at *P* < 0.05. All statistical analyses were performed in RStudio (R Core Team, 2023) with the “aov” function.

## 3 Results

### 3.1 Disease symptoms and isolation of *Neonectria*-like isolates

**Figure 2:**
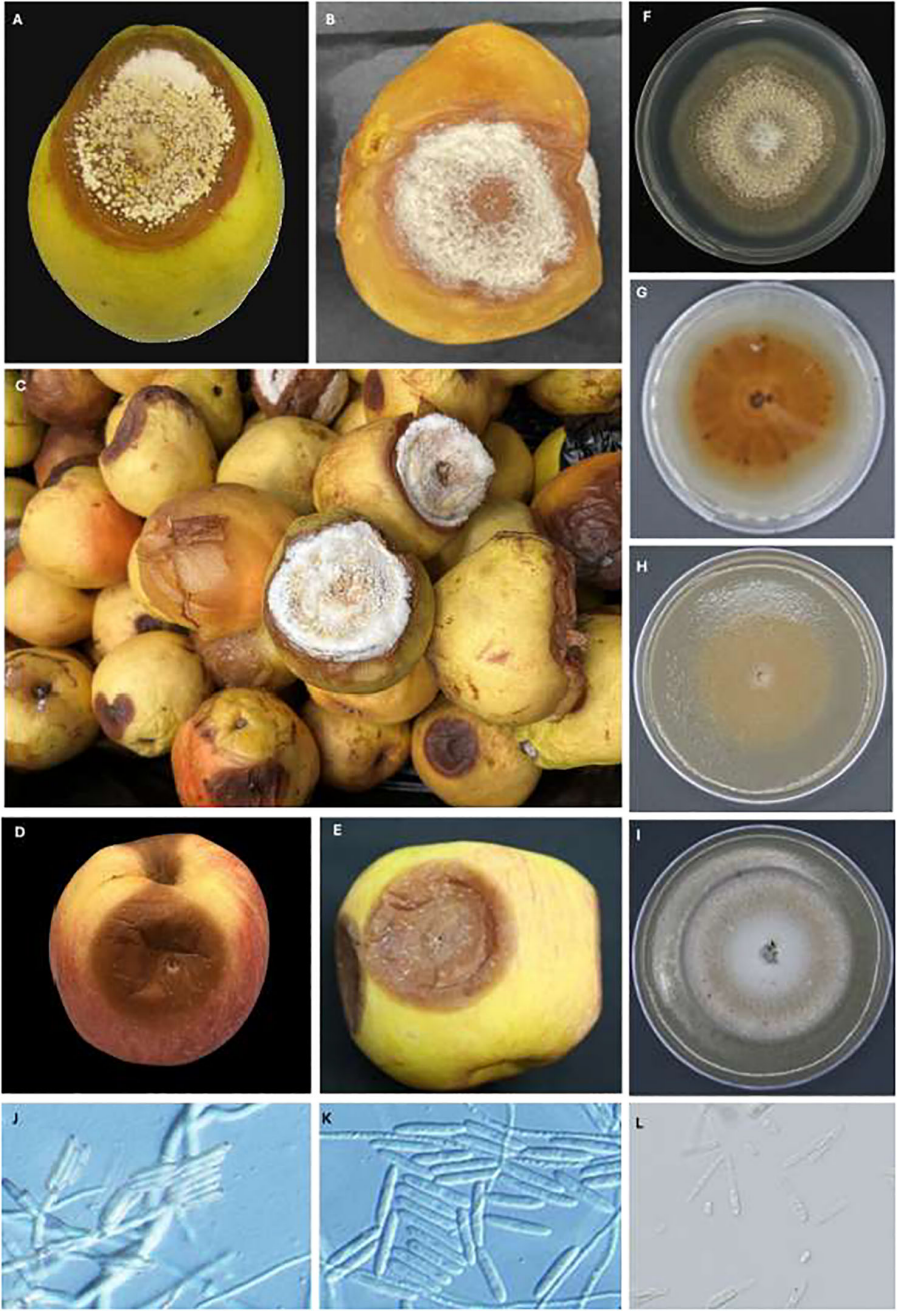
Neonectria fruit rot (NFR) caused by *Neonectria candida* on "Green d'Anjou" pear after four **(A)** and eight **(B)** months on storage in regular atmosphere at 0.5°C and advanced NFR symptoms on "Green d'Anjou" pears after 8 months **(C)**. Note the profuse sporulation on with-yellowish lesions, sunken NFR lesions on "Gala" **(D)** and "Fuji" apples **(E)** five months of storage in regular atmosphere at 1.5°C; Front **(F)** and reverse **(G)** colonies of *N. candida* isolate 17-703 grown on PDA, **(F)** on V8 **(H)** and on AJA **(I)** for 28 days at 23°C under 12 hour alternating photoperiod; sporodochia and conidiophores of *N. candida* **(J)**, macroconidia **(K)** and microconidia **(L)**. Scale bars in **(J–L)** are at 50 µm.

Symptoms first appeared as light- to dark-brown, soft, water-soaked and slightly sunken lesions (**Figures 2A, D, E**). The lesions expanded rapidly, especially on pears (**Figures 2B, C**). After four months at 0.5 °C, lesions on pear fruit were frequently blanketed by white to yellowish pustules and mycelial mats that covered the entire affected area (**Figures 2A-C**). Disease progression was slower on apple and no mycelia or sporulation was observed on ‘Gala’ and ‘Fuji’ apples stored for eight months at 1.5 °C (**Figures 2D, E**). From 2017 to 2019, 52 *Neonectria*-like isolates were obtained from multiple apple and pear packinghouses in Washington and Oregon (**Table 1**). Collections included 28, 19, and five isolates in 2017, 2018, and 2019, respectively, with 23 originating from Washington and 29 from Oregon. Pears accounted for most isolates (49; 94%), i.e., from different cultivars ‘Green d’Anjou’ (29; 59%), ‘Red d’Anjou’ (13; 27%), and ‘Bosc’ (7; 14%). Only three isolates were recovered from apples, all from the cultivar ‘Gala’ (**Table 1**). Isolates were collected from five counties in WA and three counties in OR (**Figure 1**).

On PDA, the Neonectria-like isolates produced a white colony within seven days that turned light to dark brown after 14–28 days (**Figure 2F, G**). Concentric white mycelial rings developed, and sporodochia formed after about 21 days at 23°C (**Figures 2F, G**). Conidiophores, arising laterally from individual hyphae, were 35–40 µm long. Sporodochia contained abundant conidial masses (**Figure 2J**). Macroconidia were rare, hyaline, straight to slightly curved, cylindrical to ellipsoidal, aseptate to bisepate, with rounded ends, and measured 23–30 µm (**Figures 2J, K**). Microconidia (n = 50) were hyaline, aseptate, ovoid, and 3–5 ± 1.7 µm long (**Figure 2L**). Asci, ascospores, and perithecia were not observed on PDA for up to 60 days at 23°C.

### 3.2 Multilocus sequence analysis

To obtain a general taxonomic frame of the isolates, BLAST searches in GenBank returned ≥ 99% identity with *N. candida* for 50 of 52 isolates in the *β-TUB* and *TEF1* datasets and for 51 of 52 isolates in the LSU and ITS datasets. All 52 isolates were identified as *Neonectria candida* (syn. *N. ramulariae*; anamorph *Cylindrocarpon obtusiusculum*) based on *β-TUB*, *TEF1*, LSU and ITS rDNA sequences (**Table 1**).

**Figure 3:**
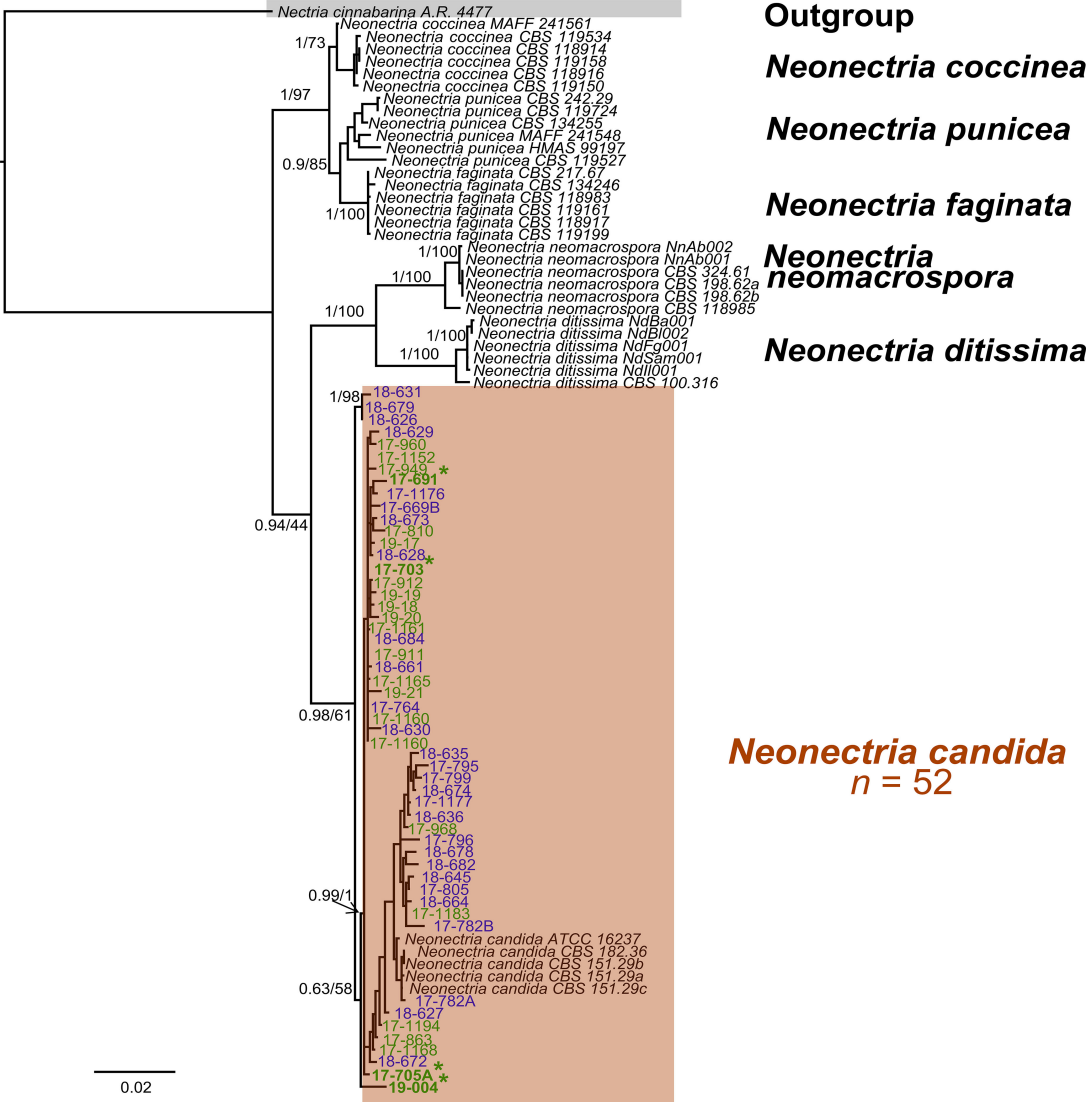
Phylogenetic tree from Bayesian inference (BI) analyses of the concatenated sequences of *β-TUB, TEF1*, LSU, and ITS rDNA from multiple *Neonectria* isolates collected from apple and pear fruit in Washington State and Oregon and other *Neonectria* reference sequences. BI posterior probabilities/Maximum likelihood bootstrap values are indicated at the branches. BI posterior probabilities >0.70 and ML bootstrap support >70% were deemed reliable. Asterisks indicate isolates from apple. Blue and green fonts indicate isolates from Oregon and Washington State, respectively.

A pairwise homogeneity test detected no significant incongruence among the four loci (*P* = 0.01). Separate Bayesian (BI) analyses of the individual genes produced slightly different topologies (**Supplementary Figures S1A–D**), but the concatenated dataset resolved all 52 pome-fruit isolates in a well-supported clade with *N. candida* reference sequences with a BI posterior probability of 0.98 (**Figure 3**). This clade was sister to a group containing *N. neomacrospora* and *N. ditissima*. Within the *N. candida* clade, two subclades were evident: one comprising 26 isolates from this study and another comprising 23 isolates plus the reference sequences (posterior probability = 0.63). Three isolates from Oregon pears (18-626, 18-631, 18-679) formed a strongly supported minor sister group to the main *N. candida* cluster. Maximum-likelihood (ML) analysis of the concatenated alignment yielded an equivalent topology, distinguishing *N. candida* from non-candida reference sequences with 61% bootstrap support (**Figure 3**), in agreement with the individual-gene ML trees (63–81% bootstrap; **Supplementary Figures S1A–D**). Pairwise ITS sequence divergence averaged 0.6–5.5% between *N. candida* and *N. ditissima* and 2.7–7.7% between *N. candida* and *N. neomacrospora* (**Table 3**). No phylogenetic structure was associated with the state of origin (Washington *vs*. Oregon) or with host (apple *vs*. pear) with isolates from all sources being intermingled throughout the *N. candida* clade.

**Table 3:** Range (minimum-maximum) of ITS-rDNA pairwise sequence divergence among *Neonectria* species.

Species	*N. candida*	*N. ditissima*	*N. neomacrospora*	*N. coccinea*	*N. punicea*	*N. faginata*
*N. candida*	0.0-2.2^a^					
*N. ditissima*	0.6-5.5	0.0-2.8				
*N. neomacrospora*	2.7-7.1	0.6-7.1	0.0-3.4			
*N. coccinea*	3.0-8.6	1.5-5.8	4.9-7.2	0.0-3.3		
*N. punicea*	3.1-8.8	1.2-4.9	4.9-7.2	0.7-3.1	0.0-1.6	
*N. faginata*	3.3-5.2	1.6-4.9	4.8-7.2	1.1-3.3	0.2-1.3	0.0-0.0

^a^Percentages < 3% indicate closely related species (Schoch et al., 2011; Nilsson et al., 2008).

### 3.3 Effect of photoperiod on growth and sporulation *in vitro*

**Figure 4:**
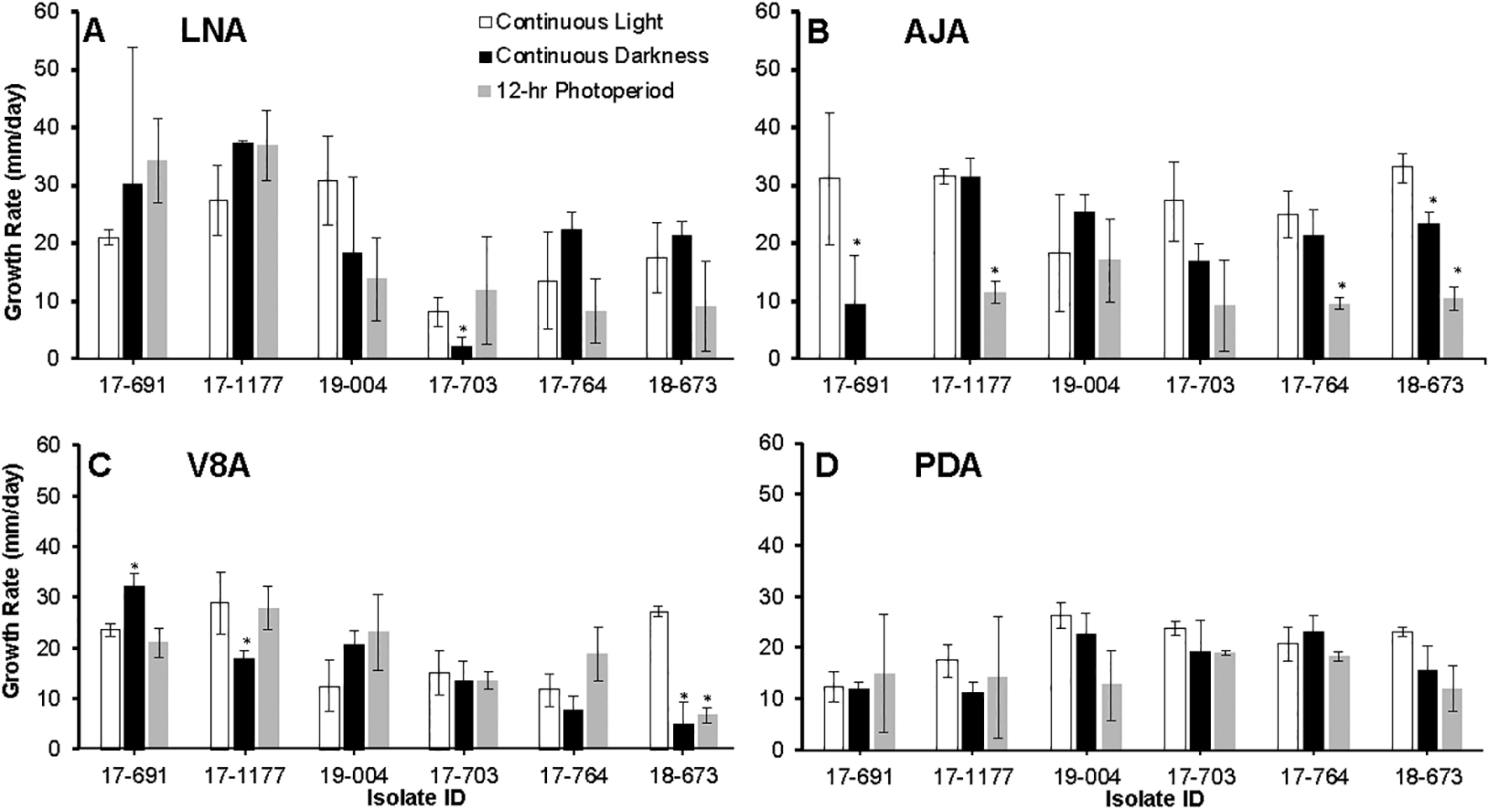
Growth rates of six *Neonectria candida* isolates on low nutrient agar (LNA, **A**), apple juice agar (AJA, **B**), V8 agar (V8A, **C**), and potato dextrose agar (PDA, **D**) for up to 21 days under continuous light, continuous darkness, and 12 hour alternating photoperiod. Data bars are the average of six values per treatment across two experimental runs. Vertical bars are standard deviations of the means. Asterisks indicate significant differences between media across isolates.

Photoperiod and isolate each had a significant effect on radial growth of *N. candida* (*P* < 0.05), whereas growth medium alone did not (*P* = 0.143). Significant interactions were detected for medium × photoperiod, medium × isolate, and the three-way combination (*P* < 0.05); the photoperiod × isolate interaction was marginal (*P* = 0.05). After 21 days in continuous light, colonies grew fastest on AJA (27.8 mm/day) and V8A (27.7 mm/day), followed by LNA (21.3 mm/day) and PDA (20.5 mm/day) (**Figure 4**). The slowest growth of 9.6 mm/day occurred on AJA under a 12 h light/12 h dark cycle (**Figure 4B**). Overall isolates 17–703 from apples grew the least on LNA, AJA and V8A, especially under a 12 hr photoperiod (**Figures 4A-C**). Photoperiod did not noticeably alter colony pigmentation on any medium. Morphology varied with medium: isolate 17–703 formed hyaline, concentrically ringed colonies on LNA, but typical brown *Neonectria* colonies with abundant conidiophores and pycnidia on PDA and especially V8A. Colonies on AJA became light to dark brown only after 28 days (**Figures 2F-I**).

**Figure 5:**
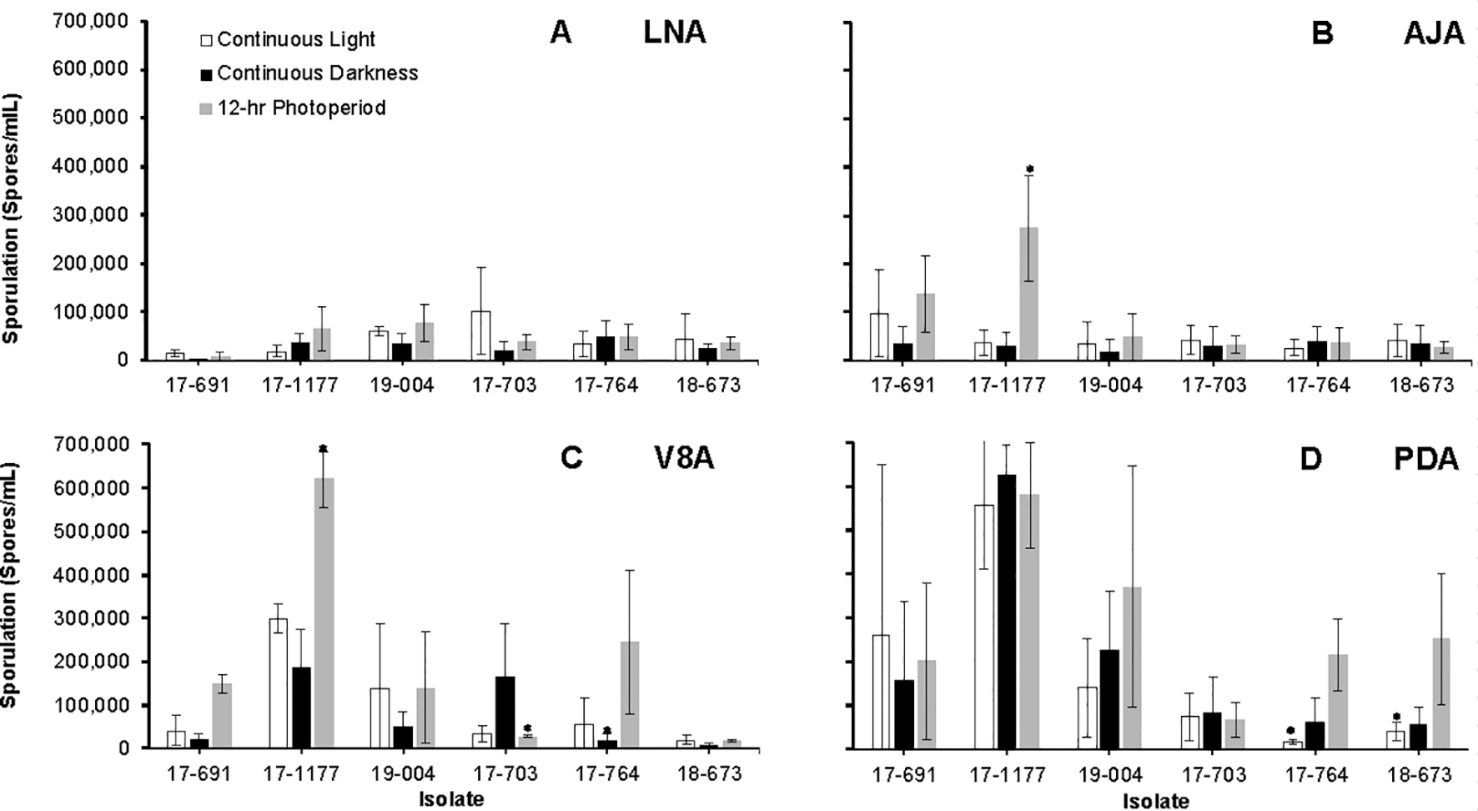
Average sporulation of six *Neonectria candida* isolates on low nutrient agar (LNA, **A**), apple juice agar (AJA, **B**), V8 agar (V8A, **C**), and potato dextrose agar (PDA, **D**) media under continuous light, continuous darkness, and a 12-hr alternating photoperiod, after 60 days of incubation at 23°C. Data bars are the average of six values per treatment across two experimental runs. Vertical bars indicate the standard deviations of the means. Asterisks indicate significant differences between media across isolates.

Sporulation differed significantly among culture media, photoperiods, isolates, and the medium × isolate interaction (*P* < 0.05). Interactions between medium × photoperiod, photoperiod × isolate, and the three-way combination were not significant (*P* > 0.05). Conidial production was significantly reduced under continuous darkness on most media but particularly on LNA and AJA (**Figures 5A, B**). Sporulation peaked on PDA and V8A under a 12 h light/12 h dark cycle (**Figures 5C, D**) with isolate 17–1177 sporulating the most.

### 3.4 Pathogenicity and virulence on apple and pear fruit

All six *N. candida* isolates were pathogenic to apples and pears at both room and cold temperatures. There was no significant effect of the host (apple *vs* pear), isolate, temperature, and all associated interactions on Neonectria Fruit Rot (NFR) incidence. In contrast, virulence, expressed as the lesion size, was strongly influenced by all these factors and all associated interactions (*P* < 0.05). For most isolates, both NFR incidence and lesion diameter were greater on pear fruit at both temperatures except for isolate 17–691 which exhibited similar virulence on both hosts (**Table 4**). On ‘Fuji’ apples, NFR incidence ranged from 56% to 91.7% after 28 days at 22 °C and from 50% to 80.5% after 120 days at 1.5°C (**Table 4**). On ‘Green d’Anjou’, NFR incidence ranged from 5.6 to 94.4 after 28 days at 22 °C and from 80.6 to 100%, after four months at 0.5°C.

**Table 4:** Incidence of Neonectria fruit rot and lesion diameter caused by *Neonectria candida* on apple and pear fruit.

Isolate	Original Host	Apple				Pear			
		1.5°C		22°C		0.5°C		22°C	
		NFR Incidence (%)^1^	Lesion diameter (mm)^2^	NFR Incidence	Lesion diameter	NFR Incidence	Lesion diameter	NFR Incidence	Lesion diameter
17-691	Apple	50.5	04.0 ± 0.2 d	56.0	04.2 ± 0.5 e	80.6	09.1 ± 0.7 d	5.6	02.5 ± 0.9 d
17-703	Apple	69.4	14.0 ± 1.4 b	88.9	19.4 ± 0.9 b	91.7	33.2 ± 1.1 a	88.9	38.2 ± 1.5 ab
19-004	Apple	72.2	08.8 ± 0.7 c	91.7	30.3 ± 1.4 a	100.0	27.5 ± 0.8 b	77.8	34.4 ± 1.9 c
17-764	Pear	80.5	14.2 ± 1.5 b	58.3	11.5 ± 1.2 d	97.2	39.7 ± 0.7 a	86.1	41.3 ± 1.8 a
17-1177	Pear	63.9	70.1 ± 0.8 a	66.7	16.1 ± 1.5 c	91.2	31.0 ± 1.0 c	88.9	36.5 ± 1.5 b
18-673	Pear	69.4	15.7 ± 1.7 b	67.8	16.4 ± 1.4 c	100.0	40.6 ± 0.4 a	94.4	44.2 ± 1.3 a

^1,2^ Incidence of Neonectria Fruit Rot (NFR) and lesion diameter (mm) were measured after 28 and 120 days of incubation at 22°C and 1.5°C on Fuji apples and 22°C and 0.5°C on Green d’Anjou pears, respectively. Data are the means ± standard deviations of 36 fruit for four replications of nine fruit per isolate and host. Values within the same column followed by different letters are significantly different based on ANOVA and Student’s t test at P < 0.05.

## 4 Discussion

*Neonectria ditissima* and *N. punicea* have previously been reported as causal agents of NFR on apples (Xu and Robinson, 2010; Wu et al., 2022; Wesche and Weber, 2023), while *N. candida* Wollenw (syn. *N. ramulariae*, anamorph *Cylindrocarpon obtusiusculum*) was associated with NFR on pears (Wenneker et al., 2016). In this study, Bayesian and Maximum Likelihood multilocus sequence analyses (MLSA) based on four concatenated and individual loci confirmed that all 52 *Neonectria* isolates collected from decayed apple and pear fruit clustered within a well-supported clade containing *N. candida* reference sequences, including isolates from *Malus sylvestris* from the UK and Portugal. This is the second report of *N. candida* causing NFR on pear following its initial report in the Netherlands (Wenneker et al., 2016) and represents the first report of *N. candida* as the causal agent of NFR on apple fruit globally. Bayesian phylogenetic analysis placed the *N. candida* clade as a sister group to clades containing *N. ditissima* and *N. neomacrospora*, while *N. punicea*, the third species previously linked to NFR on apples, was more distantly related. These relationships were further supported by pairwise ITS rDNA sequence divergence, which reached 5.5% between *N. candida* and *N. ditissima*, 7.1% with *N. neomacrospora*, and 8.1% with *N. punicea*.

The ecology and epidemiology of *N. candida* remain largely unresolved. First described as a root pathogen of alfalfa (*Medicago sativa*) (Cormack, 1937), the species has since been linked to cankers on almond seedlings (*Prunus amygdalus*) (Marek et al., 2013), bark cankers of beech in North America (Castlebury et al., 2006), beech seed rot in Japan (Hirooka et al., 2012), and postharvest decay of pear fruit in the Netherlands (Wenneker et al., 2016). Beyond its role as a plant pathogen, *N. candida* is considered a prominent colonizer of topsoil and leaf litter in European beech forests (Jankowiak et al., 2016) and a widespread soilborne fungus worldwide (Booth, 1966; Domsch et al., 2007). Whether potential reservoir hosts, such as American or Japanese beech, occur near the commercial pear and apple orchards surveyed in this study is unknown. Nevertheless, the fungus may persist in orchard soils or overwinter in surface litter, providing inoculum for the following season. Central Washington orchards commonly use sprinkler irrigation, which could splash *N. candida* conidia from the soil onto fruit, especially those low in the canopy. Wenneker et al. (2016) noted heavy soil contamination on pears with NFR in the Netherlands, underscoring the likely epidemiological role of soil and the value of orchard floor and bin sanitation in disease management. Moreover, although *N. candida* can induce cankers on almonds and beech (Castlebury et al., 2006; Marek et al., 2013), its capacity to produce cankers or dieback on apple and pear trees, and thereby generate primary inoculum for NFR, has not been studied in the PNW. In our study, *in vitro* cultures and inoculated fruit were observed for up to four months of cold storage, yet no perithecia with asci and ascospores developed. Determining whether the teleomorph occurs in regional orchards, and whether sexual reproduction contributes to the fungus’ life cycle, remains an important avenue for future research.

Both light regime and nutrient sources (agar media) significantly influenced the growth and sporulation of *N. candida*. Continuous light supported the most vigorous growth, followed by a 12-hour light/dark photoperiod, while continuous darkness was the least favorable. Among the media tested, V8A and LNA supported greater colony expansion compared to AJA or PDA. Across all lighting conditions, colonies on PDA developed yellowish to brown pigmentation after 14 days, consistent with previous observations (Lombard et al., 2015; Wenneker et al., 2016). Jankowiak et al. (2016), however, reported yellow to orange pigmentation under continuous darkness on PDA. Unlike *N. ditissima*, which requires specific light and media combinations to induce sporulation (Scheper et al., 2014), *N. candida* sporulated readily under all light regimes and on all media types tested, although it was more abundant under the 12-hour photoperiod on PDA and V8A. Conidia produced on PDA were predominantly two-septate, differing from the mostly one-septate conidia described by Jankowiak et al. (2016). While more isolates should be examined to confirm diagnostic traits, the conidia of *N. candida*, typically straight to ellipsoidal and approximately 30 µm long, may help distinguish this species morphologically from other NFR pathogens. In contrast, *N. ditissima* and *N. punicea* produce longer, crescent-shaped conidia ranging from 35 to 45 µm (Castlebury et al., 2006; Wu et al., 2022). Moreover, of the six isolates tested, only one (17–703 from apples) produced macro-conidia and did not cause significantly different NFR incidence, suggesting that micro and macroconidia of *N. candida* can equally infect pome fruit in contrast with *N. ditissima* (Wesche and Weber, 2023).

Pathogenicity tests conducted on detached, wounded, and artificially inoculated ‘Fuji’ apples and ‘Green d’Anjou’ pears showed that *N. candida* isolates from both apple and pear were pathogenic to fruit of both hosts. Infections produced similar symptoms across hosts: soft, brown lesions, while white to yellowish mycelia were mostly seen on pears. These findings on ‘Green d’Anjou’ pears are consistent with those previously reported for ‘Conference’ pears in the Netherlands (Wenneker et al., 2016) and confirms the pathogenicity of *N. candida* on apple fruit. Despite the difference in host origin, no evidence of host specificity was observed. However, broader screening across additional apple and pear cultivars is needed to fully evaluate host susceptibility. Notably, ‘Green d’Anjou’ and ‘Gala’ are the most widely planted pear and apple cultivars, respectively, in the PNW. Their demonstrated susceptibility to *N. candida* highlights a potential emerging pathogen to postharvest fruit quality in the region. In comparison, susceptibility of apple cultivars to *N. ditissima*, the causal agent of European canker, varies among apple cultivars with ‘Gala’, ‘Braeburn’, and ‘Red Delicious’ being the most susceptible, while ‘Golden Delicious’ and ‘Honeycrisp’ show reduced susceptibility (Dubin and English, 1974; Xu and Robinson, 2010; Gómez-Cortecero et al., 2016; Alves and Nunes, 2017). Whether such differences in susceptibility extend to fruit infections by *N. candida* remain unknown.

While pathogenicity studies of *N. candida* on other plant hosts are limited, previous research has documented 30% infection incidence in beech seeds (Hirooka et al., 2012) and a 0.2% incidence in beech litter (Jankowiak et al., 2016). In our study, isolates from pear were generally more virulent than those from apples, with disease incidence on pears ranging from 86.1% to 100%, compared to a broader range of 5.6% to 100% for apples. Notably, none of the apple isolates were obtained from ‘Fuji’, the cultivar used in pathogenicity assays, indicating a need for future virulence testing across additional apple cultivars. The ability of *N. candida* to cause cankers or shoot dieback on pome fruit trees remains untested. Given its association with cankers on beech trees (Castlebury et al., 2006), such potential should be investigated to clarify the pathogen’s epidemiology.

## 5 Conclusions

This study identifies *N. candida* as the causal agent of Neonectria fruit rot (NFR) in pear and documents the first confirmed case of NFR in apple globally. The pathogen was detected throughout the Pacific Northwest, from northern Washington to southern Oregon, demonstrating its capacity to thrive across diverse environmental conditions. Additional work is needed to clarify its etiology and epidemiology and to evaluate its sensitivity to current pre- and postharvest fungicides. Such research will be essential for designing effective management strategies that can curb future outbreaks and protect fruit quality.

## Data Availability

The datasets presented in this study can be found in online repositories. The names of the repository/repositories and accession number(s) can be found in the article/**Supplementary Material**.
