# Supplementary material for: Morpho-phylogenic characterization of Neonectria candida as a causal agent of a postharvest rot of pome fruit in the U.S. Pacific Northwest
Source: Front Plant Sci. 2025 Sep 11;16:1661560. doi: 10.3389/fpls.2025.1661560 (PMC12460409; doi:10.3389/fpls.2025.1661560)
Supplement: Supplementary file 1 [file DataSheet1.pdf]

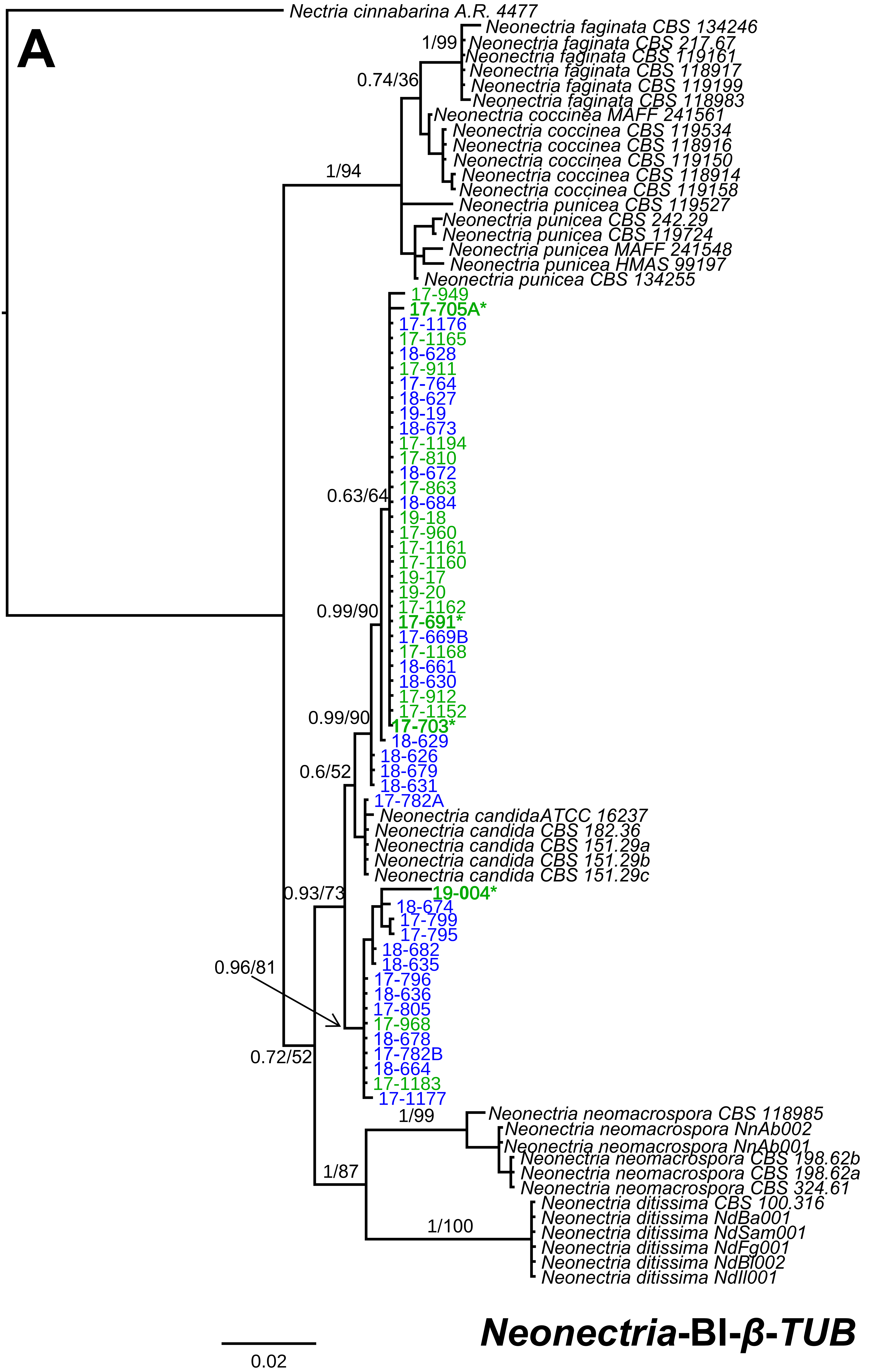

A
Nectria cinnabarina A.R. 4477
1/99
Neonectria faginata CBS 134246
Neonectria faginata CBS 217.67
Neonectria faginata CBS 119161
Neonectria faginata CBS 118917
Neonectria faginata CBS 119199
Neonectria faginata CBS 118983
0.74/36
Neonectria coccinea MAFF 241561
Neonectria coccinea CBS 119534
Neonectria coccinea CBS 118916
Neonectria coccinea CBS 119150
Neonectria coccinea CBS 118914
Neonectria coccinea CBS 119158
1/94
Neonectria punicea CBS 119527
Neonectria punicea CBS 242.29
Neonectria punicea CBS 119724
Neonectria punicea MAFF 241548
Neonectria punicea HMAS 99197
Neonectria punicea CBS 134255
17-949
17-705A*
17-1176
17-1165
18-628
17-911
17-764
18-627
19-19
18-673
17-1194
17-810
18-672
17-863
0.63/64
18-684
19-18
17-960
17-1161
17-1160
19-17
19-20
17-1162
0.99/90
17-691*
17-669B
17-1168
18-661
18-630
17-912
17-1152
0.99/90
17-703*
18-629
18-626
0.6/52
18-679
18-631
17-782A
Neonectria candidaATCC 16237
Neonectria candida CBS 182.36
Neonectria candida CBS 151.29a
Neonectria candida CBS 151.29b
Neonectria candida CBS 151.29c
0.93/73
19-004*
18-674
17-799
17-795
18-682
0.96/81
18-635
17-796
18-636
17-805
17-968
18-678
0.72/52
17-782B
18-664
17-1183
17-1177
1/99
Neonectria neomacrospora CBS 118985
Neonectria neomacrospora NnAb002
Neonectria neomacrospora NnAb001
Neonectria neomacrospora CBS 198.62b
1/87
Neonectria neomacrospora CBS 198.62a
Neonectria neomacrospora CBS 324.61
Neonectria ditissima CBS 100.316
Neonectria ditissima NdBa001
1/100
Neonectria ditissima NdSam001
Neonectria ditissima NdFg001
Neonectria ditissima NdBl002
Neonectria ditissima NdIl001
Neonectria-BI-β-TUB
0.02

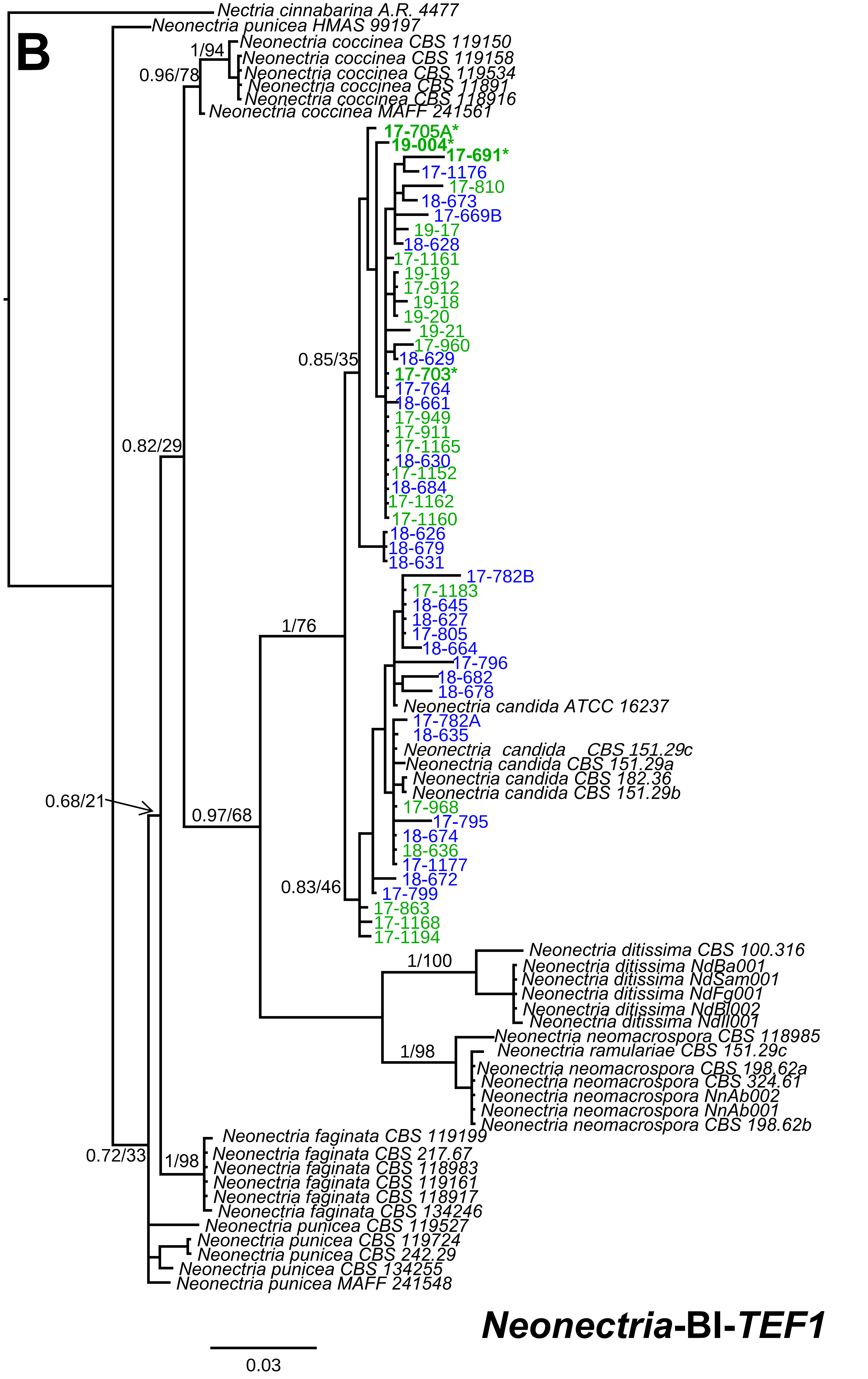

B
Nectria cinnabarina A.R. 4477
Neonectria punicea HMAS 99197
1/94
Neonectria coccinea CBS 119150
Neonectria coccinea CBS 119158
0.96/78
Neonectria coccinea CBS 119534
Neonectria coccinea CBS 11891
Neonectria coccinea CBS 118916
Neonectria coccinea MAFF 241561
17-705A*
19-004*
17-691*
17-1176
17-810
18-673
17-669B
19-17
18-628
17-1161
19-19
17-912
19-18
19-20
19-21
17-960
18-629
0.85/35
17-703*
17-764
18-661
17-949
17-911
0.82/29
17-1165
18-630
17-1152
18-684
17-1162
17-1160
18-626
18-679
18-631
17-782B
17-1183
18-645
18-627
1/76
17-805
18-664
17-796
18-682
18-678
Neonectria candida ATCC 16237
17-782A
18-635
Neonectria candida CBS 151.29c
Neonectria candida CBS 151.29a
Neonectria candida CBS 182.36
Neonectria candida CBS 151.29b
0.68/21
17-968
0.97/68
17-795
18-674
18-636
17-1177
18-672
0.83/46
17-799
17-863
17-1168
17-1194
Neonectria ditissima CBS 100.316
1/100
Neonectria ditissima NdBa001
Neonectria ditissima NdSam001
Neonectria ditissima NdFg001
Neonectria ditissima NdBl002
Neonectria ditissima NdIl001
Neonectria neomacrospora CBS 118985
1/98
Neonectria ramulariae CBS 151.29c
Neonectria neomacrospora CBS 198.62a
Neonectria neomacrospora CBS 324.61
Neonectria neomacrospora NnAb002
Neonectria neomacrospora NnAb001
Neonectria neomacrospora CBS 198.62b
Neonectria faginata CBS 119199
0.72/33
Neonectria faginata CBS 217.67
1/98
Neonectria faginata CBS 118983
Neonectria faginata CBS 119161
Neonectria faginata CBS 118917
Neonectria faginata CBS 134246
Neonectria punicea CBS 119527
Neonectria punicea CBS 119724
Neonectria punicea CBS 242.29
Neonectria punicea CBS 134255
Neonectria punicea MAFF 241548
Neonectria-BI-TEF1
0.03

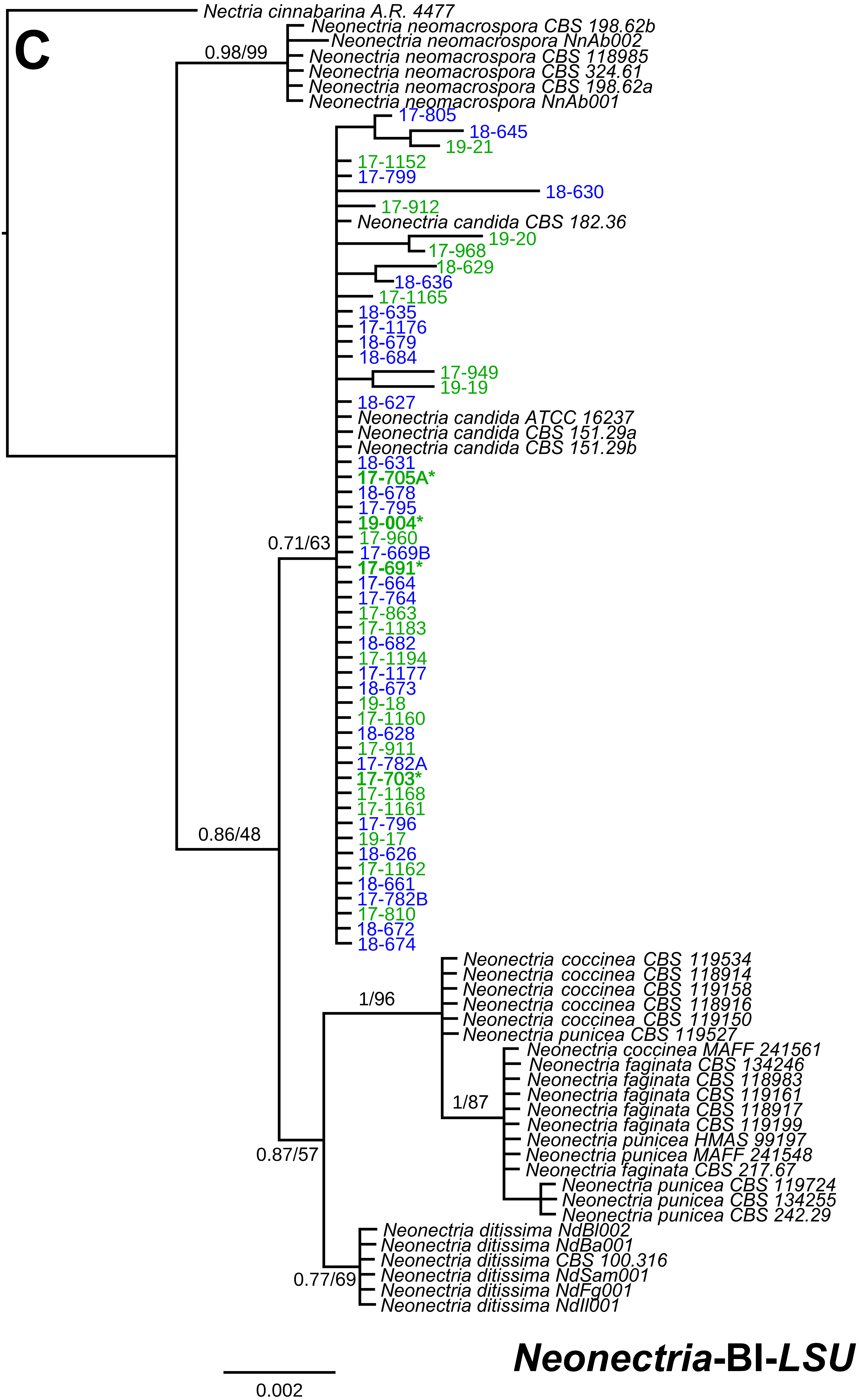
C
Nectria cinnabarina A.R. 4477
0.98/99
Neonectria neomacrospora CBS 198.62b
Neonectria neomacrospora NnAb002
Neonectria neomacrospora CBS 118985
Neonectria neomacrospora CBS 324.61
Neonectria neomacrospora CBS 198.62a
Neonectria neomacrospora NnAb001
17-805
18-645
19-21
17-1152
17-799
18-630
17-912
Neonectria candida CBS 182.36
19-20
17-968
18-629
18-636
17-1165
18-635
17-1176
18-679
18-684
17-949
19-19
18-627
Neonectria candida ATCC 16237
Neonectria candida CBS 151.29a
Neonectria candida CBS 151.29b
18-631
17-705A*
18-678
17-795
19-004*
0.71/63
17-960
17-669B
17-691*
17-664
17-764
17-863
17-1183
18-682
17-1194
17-1177
18-673
19-18
17-1160
18-628
17-911
17-782A
17-703*
17-1168
17-1161
17-796
0.86/48
19-17
18-626
17-1162
18-661
17-782B
17-810
18-672
18-674
Neonectria coccinea CBS 119534
Neonectria coccinea CBS 118914
Neonectria coccinea CBS 119158
1/96
Neonectria coccinea CBS 118916
Neonectria coccinea CBS 119150
Neonectria punicea CBS 119527
Neonectria coccinea MAFF 241561
Neonectria faginata CBS 134246
Neonectria faginata CBS 118983
Neonectria faginata CBS 119161
1/87
Neonectria faginata CBS 118917
Neonectria faginata CBS 119199
Neonectria punicea HMAS 99197
0.87/57
Neonectria punicea MAFF 241548
Neonectria faginata CBS 217.67
Neonectria punicea CBS 119724
Neonectria punicea CBS 134255
Neonectria punicea CBS 242.29
Neonectria ditissima NdBl002
Neonectria ditissima NdBa001
Neonectria ditissima CBS 100.316
0.77/69
Neonectria ditissima NdSam001
Neonectria ditissima NdFg001
Neonectria ditissima NdIl001
0.002
Neonectria-BI-LSU

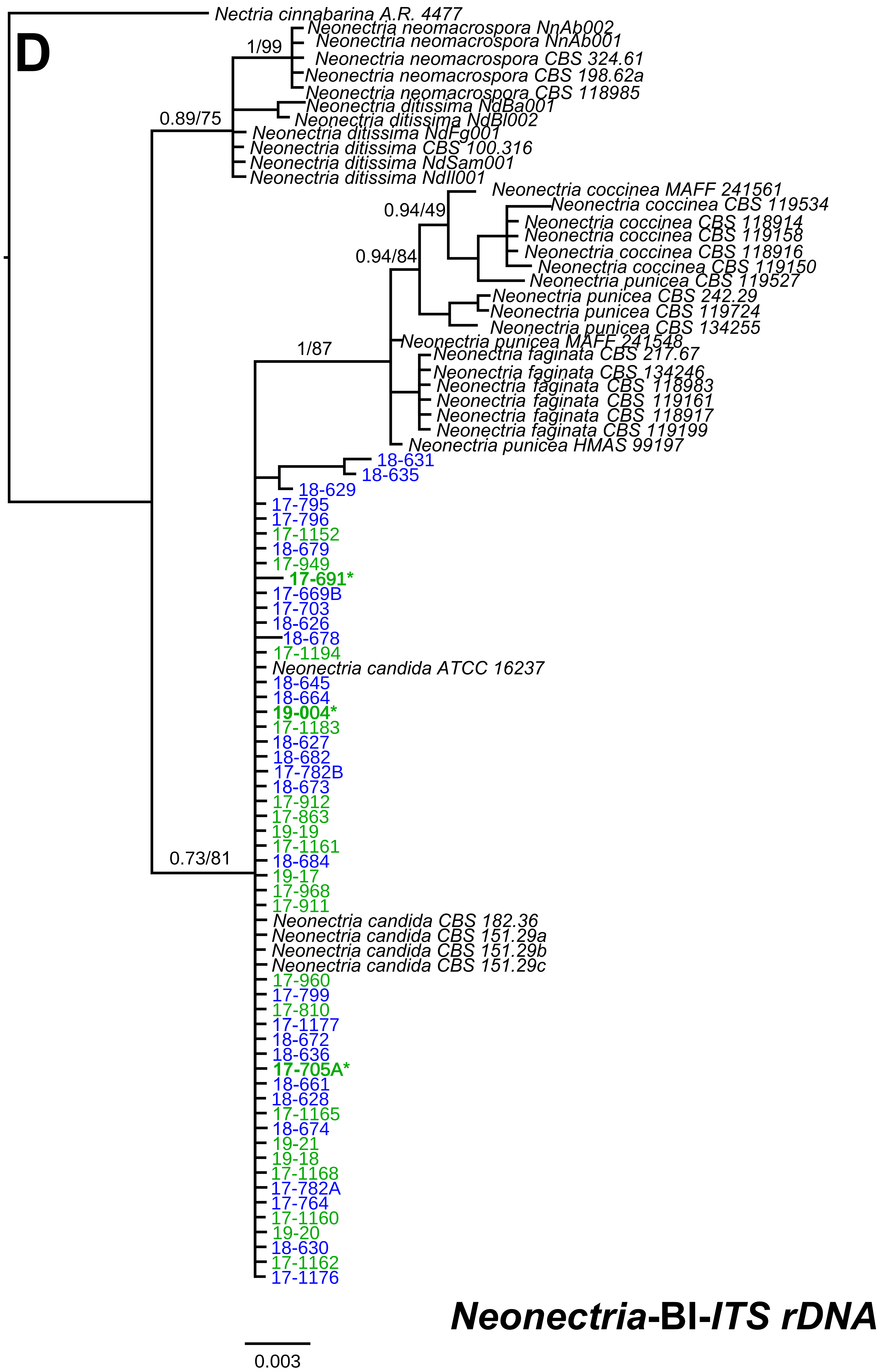
D
Nectria cinnabarina A.R. 4477
1/99
Neonectria neomacrospora NnAb002
Neonectria neomacrospora NnAb001
Neonectria neomacrospora CBS 324.61
Neonectria neomacrospora CBS 198.62a
Neonectria neomacrospora CBS 118985
0.89/75
Neonectria ditissima NdBa001
Neonectria ditissima NdBl002
Neonectria ditissima NdFg001
Neonectria ditissima CBS 100.316
Neonectria ditissima NdSam001
Neonectria ditissima NdIl001
0.94/49
Neonectria coccinea MAFF 241561
Neonectria coccinea CBS 119534
Neonectria coccinea CBS 118914
Neonectria coccinea CBS 119158
Neonectria coccinea CBS 118916
Neonectria coccinea CBS 119150
0.94/84
Neonectria punicea CBS 119527
Neonectria punicea CBS 242.29
Neonectria punicea CBS 119724
Neonectria punicea CBS 134255
1/87
Neonectria punicea MAFF 241548
Neonectria faginata CBS 217.67
Neonectria faginata CBS 134246
Neonectria faginata CBS 118983
Neonectria faginata CBS 119161
Neonectria faginata CBS 118917
Neonectria faginata CBS 119199
Neonectria punicea HMAS 99197
18-631
18-635
18-629
17-795
17-796
17-1152
18-679
17-949
17-691*
17-669B
17-703
18-626
18-678
17-1194
Neonectria candida ATCC 16237
18-645
18-664
19-004*
17-1183
18-627
18-682
17-782B
18-673
17-912
17-863
19-19
17-1161
0.73/81
18-684
19-17
17-968
17-911
Neonectria candida CBS 182.36
Neonectria candida CBS 151.29a
Neonectria candida CBS 151.29b
Neonectria candida CBS 151.29c
17-960
17-799
17-810
17-1177
18-672
18-636
17-705A*
18-661
18-628
17-1165
18-674
19-21
19-18
17-1168
17-782A
17-764
17-1160
19-20
18-630
17-1162
17-1176
Neonectria-BI-ITS rDNA
0.003

Supplementary Figure 1. Phylogenetic trees from Bayesian analyses of the *β-TUB* (**A**), *TEF1* (**B**), *LSU* (**C**), and *ITS rDNA* (**D**) genes from multiple *Sarocladium* isolates collected from apple and pear in Washington and Oregon and other *Sarocladium* and *Acremonium* references' sequences. BI posterior probabilities/ML bootstrap values are indicated at branches. Bayesian inference posterior probabilities above 0.70 and ML bootstrap support values above 70% were deemed reliable. Asterisks indicate isolates from apple. Blue and green fonts indicate isolates collected from Oregon and Washington State, respectively.
