# Supplementary material for: Morpho-phylogenic characterization of Neonectria candida as a causal agent of a postharvest rot of pome fruit in the U.S. Pacific Northwest
Source: Front Plant Sci. 2025 Sep 11;16:1661560. doi: 10.3389/fpls.2025.1661560 (PMC12460409; doi:10.3389/fpls.2025.1661560)
Supplement: Supplementary file 2 [file DataSheet2.pdf]

Supplementary Table 1

Accession numbers of *Neonectria* reference sequences used in phylogenetic analyses. Sequences were acquired from NCBI. Host species and location are provided, where available. Asterisk indicates the outgroup reference sequence.

					GenBank Accession No.			
**Species**	**Strain ID**	**Isolate Synonym**	**Host**	**Origin**	***ß-TUB***	***TEF1***	***ITS***	***LSU***
**Nectria cinnabarina*	A.R. 4477	-	*Aesculus sp.*	France	HM484606	HM484527	HM484548	HM484562
Neonectria coccinea	CBS 119534	-	*Fagus sylvatica*	France	KC660723	KC660436	KC660501	KC660619
Neonectria coccinea	CBS 118914	-	*Fagus sylvatica*	France	KC660725	KC660435	KC660500	KC660607
Neonectria coccinea	CBS 119158	-	*Fagus sp.*	Germany	KC660727	JF268734	KC660521	KC660620
Neonectria coccinea	MAFF 241561	-	*Fagus crenata*	Japan	KC660729	KC660493	KC660548	KC660552
Neonectria coccinea	CBS 118916	-	*Fagus sylvatica*	Romania	KC660719	KC660442	KC660505	KC660601
Neonectria coccinea	CBS 119150	-	*Fagus sylvatica*	Slovakia	KC660717	KC660440	KC660504	KC660577
Neonectria ditissima	CBS 100.316	*Neonectria galligena*	*Malus domestica*	Netherlands	HM352864	HM364350	HM364298	HM364311
Neonectria ditissima	NdBa001	*Neonectria galligena*	*Betula alleghaniensis*	USA	MT502665	MT502636	MT478954	MT482764
Neonectria ditissima	NdSam001	*Neonectria galligena*	*Sorbus americana*	USA	MT502666	MT502637	MT478955	MT482765
Neonectria ditissima	NdFg001	*Neonectria galligena*	*Fagus grandifola*	USA	MT502674	MT502645	MT478963	MT482772
Neonectria ditissima	NdBl002	*Neonectria galligena*	*Betula lenta*	USA	MT502670	MT502641	MT478959	MT482769
Neonectria ditissima	NdIl001	*Neonectria galligena*	*Ilex mucronata*	USA	MT502669	MT502640	MT478958	MT482768
Neonectria faginata	CBS 217.67	-	*Fagus grandifola*	Canada	JF268730	JF268746	HQ840385	HQ840382
Neonectria faginata	CBS 134246	-	*Fagus grandifola*	USA	KC660743	KC660457	KC660519	KC660600
Neonectria faginata	CBS 118983	-	*Fagus sp.*	USA	KC660741	KC660433	KC660498	KC660571
Neonectria faginata	CBS 119161	-	*Fagus grandifola*	USA	KC660736	KC660447	KC660509	KC660557
Neonectria faginata	CBS 118917	-	*Fagus grandifola*	USA	KC660735	KC660451	KC660513	KC660562
Neonectria faginata	CBS 119199	-	*Fagus sylvatica*	USA	KC660740	KC660455	KC660517	KC660555
Neonectria neomacrospora	CBS 324.61	-	*Abies concolor*	Netherlands	DQ789875	HM364352	JF735312	HM364318
Neonectria neomacrospora	CBS 198.62a	-	*Abies concolor*	Germany	DQ789866	JF735788	AJ009255	MH869729
Neonectria neomacrospora	CBS 198.62b	-	*Abies concolor*	Netherlands	HM352865	HM364351	-	HM364316
Neonectria neomacrospora	NnAb002	-	*Abies fraseri*	USA	MT502684	MT502653	MT478973	MT482781
Neonectria neomacrospora	NnAb001	-	*Abies fraseri*	USA	MT502685	MT502654	MT478974	MT482782
Neonectria neomacrospora	CBS 118985	-	*Tsuga heterophylla*	Canada	DQ789890	JF268755	HQ840389	HQ840380
Neonectria punicea	CBS 119724	-	*Frangula alnus*	Austria	DQ789824	KC660431	KC660496	KC660568
Neonectria punicea	CBS 119527	-	*Rhamnus fallax*	Austria	DQ789854	KC660453	KC660515	KC660575
Neonectria punicea	HMAS 99197	-	Twigs of a dicotyledon tree	China	KC660714	JF268735	KC660531	KC660558
Neonectria punicea	CBS 134255	-	Hardwood tree	France	KC660701	KC660469	KC660529	KC660598
Neonectria punicea	CBS 242.29	-	*Rhamnus sp.*	Germany	DQ789873	DQ789730	KC660522	KC660565
Neonectria punicea	MAFF 241548	-	Twigs	Japan	KC660713	KC660484	KC660541	KC660569
Neonectria ramulariae	CBS 151.29a	*Neonectria candida*	*Malus sylvestris*	UK	DQ789863	DQ789723	AY677291	HM042436
Neonectria ramulariae	CBS 151.29b	*Neonectria candida*	*Malus sylvestris*	Portugal	JF735438	JF735791	JF735313	-
Neonectria ramulariae	CBS 151.29c	*Neonectria candida*	Culture collection	China	HM054124	HM054091	HM054150	HM042436
Neonectria ramulariae	ATCC 16237	*Neonectria candida*	Culture collection	USA	HM352863	HM364349	HM364297	HM364310
Neonectria ramulariae	CBS 182.36	*Neonectria candida*	Culture collection	Netherlands	JF735439	JF735792	MH855762	MH867276
